# Editorial: Decoding the mito-immune axis: impact of mitochondria on immune regulation and pathogen defense

**DOI:** 10.3389/fcimb.2026.1935097

**Published:** 2026-08-04

**Authors:** Manmohan Kumar, Nidhi Srivastava, Shibnath Mazumder

**Affiliations:** 1 Department of Microbiology, Immunology and Molecular Genetics, University of Texas Health San Antonio, University of Texas San Antonio, San Antonio, TX, United States; 2 Department of Zoology, School of Basic and Applied Sciences, Maharaja Agrasen University, Solan, Himachal Pradesh, India; 3 Immunobiology Laboratory, Department of Zoology, University of Delhi, Delhi, India

**Keywords:** metabolic reprogramming, mitochondria in immune surveillance, mitochondria in infection, mitochondrial metabolism, mtDAMPs

## Introduction

Over the past decade, mitochondria have emerged as central regulators of host immunity, extending well beyond their canonical role in cellular bioenergetics. As key orchestrators of cellular immunometabolism, mitochondria integrate metabolic status with immune signaling pathways to regulate both innate and adaptive immune responses. In addition to providing the metabolic support required for immune cell activation and function, mitochondria actively participate in immune signaling through the production and release of mitochondrial-derived danger-associated molecular patterns (mtDAMPs) such as mitochondrial reactive oxygen species (mtROS), mitochondrial DNA (mtDNA), ATP, mitochondrial Transcription factor A (TFAM) and N-formyl peptide (NFP) (Nakahira et al., 2015). As endogenous danger signals, mtDAMPs engage pattern recognition receptors (PRRs) and trigger downstream signaling pathways that culminate in the production of pro-inflammatory mediators and the activation of innate immune responses against pathogenic infection. As central hubs of antimicrobial immunity, mitochondria are also frequent targets of pathogen-encoded virulence factors that reprogram mitochondrial functions to dampen host immune responses and facilitate infection. In this context, Yang et al. provide a comprehensive overview of the emerging role of mitochondria in host–pathogen interactions, highlighting their pivotal role as determinants of cellular metabolism and immune defense. They described how diverse pathogens manipulate mitochondrial dynamics, bioenergetics, and signaling pathways to evade antimicrobial responses, reprogram host metabolism, and establish persistent infection. Importantly, mitochondrial dysfunction resulting from these interactions serves as a potent trigger for innate immune pathways, particularly cGAS–STING signaling and NLRP3 inflammasome activation, through the release of mDAMPs. While activation of these pathways is crucial for pathogen clearance, prolonged or dysregulated engagement may drive excessive inflammation *via* inflammatory cytokines, leading to tissue injury.

Mitochondrial dysfunction is increasingly recognized as a critical driver of immune activation, linking alterations in mitochondrial homeostasis to the initiation, amplification, and persistence of inflammatory responses (Kim et al., 2025). Persistent inflammation poses a major challenge during infection. Okine et al. provide a comprehensive insight into how persistent inflammation remains a major clinical concern in HIV infection despite the widespread use of effective antiretroviral therapy (ART), contributing to ongoing immune dysregulation, an increased risk of non-AIDS comorbidities, and suboptimal long-term health outcomes. Defects in mitochondrial bioenergetics and cellular metabolism can promote the activation of inflammasomes and alter purinergic signaling pathways, resulting in sustained production of inflammatory cytokines and constant immune system stimulation. This mito–immune axis provides a mechanistic framework through which mitochondrial stress contributes to long-term immunological dysfunction and the development of cardiovascular, metabolic, and neurocognitive comorbidities commonly observed in HIV patients. This review supports the concept that therapeutic approaches aimed at improving mitochondrial function *via* purinergic antagonists and modulating downstream inflammatory pathways may complement antiviral treatment by restoring immune-metabolic balance and reducing the risk of chronic disease progression. Collectively, these reviews position mitochondria at the nexus of infection, metabolic reprogramming, and inflammatory pathology, underscoring the therapeutic potential of targeting mitochondrial pathways to restore immune homeostasis, enhance host defense, and mitigate infection-associated inflammatory damage.

The activation of immune cells is tightly coupled to metabolic reprogramming, involving glycolysis, the tricarboxylic acid (TCA) cycle, fatty acid oxidation (FAO), amino acid metabolism, and the pentose phosphate pathway (PPP) (Malla et al., 2025). Among these, glycolysis, the TCA cycle, and FAO are critical for regulating immune cell activation and functional differentiation. This metabolic plasticity supports macrophage polarization as well as B- and T-cell differentiation in response to environmental and pathogenic cues (Phan et al., 2017). The study conducted by Sun et al. demonstrated that glycolysis-derived lactate is now recognized as more than a metabolic byproduct and a biomarker of disease severity. It functions as a signaling metabolite that drives histone and protein lactylation, linking metabolic reprogramming to epigenetic and post-translational regulation of gene expression. Lactylation has emerged as a key mechanism governing macrophage polarization and the transition between pro-inflammatory and reparative phenotypes, thereby influencing immune homeostasis and the pathogenesis of sepsis. Their review highlights the intimate crosstalk between metabolism and immunity and identifies lactate-driven signaling and lactylation as promising therapeutic targets for restoring macrophage function and improving outcomes in sepsis.

Recent evidence has progressively highlighted the involvement of mitochondrial dysfunction in various diseases such as intestinal bowel disease (IBD) (Wang et al., 2025), neurodegenerative disease (Klemmensen et al., 2024), pancreatitis (Chen et al., 2024), insulin resistance (Montgomery and Turner, 2015) and autoimmunity (Ma et al., 2025).

The study conducted by Lee et al., demonstrated that the mtSTAT3 functions as a critical molecular bridge between mitochondrial metabolism and immune regulation in IBD. Enhanced mtSTAT3 activity restores mitochondrial respiratory capacity, reduces oxidative stress, suppresses pro-inflammatory and pro-fibrotic signaling, and promotes favorable signaling and alterations in the gut microbiota, collectively preserving intestinal homeostasis and attenuating disease severity. In endometriosis, Wang et al. demonstrated that mitochondrial metabolic dysregulation contributes to aberrant cellular survival, inflammatory activation, and metabolic adaptation. Mitochondria-associated genes, such as glycerol-3-phosphate dehydrogenase 2 (GPD2) and mitochondrial ribosomal protein S6 (MRPS6), have emerged as key regulators of mitochondrial bioenergetics and protein synthesis, linking altered mitochondrial function to the inflammatory microenvironment that supports progression. In case of pancreatitis, Xiaozhou et al. demonstrated that defective mitophagy compromises mitochondrial quality control, thereby driving mitochondrial dysfunction, oxidative stress, and sustained macrophage-mediated inflammatory responses. Mitogen-activated protein kinase 14 (MAPK14) has been identified as a central regulator linking mitophagy to innate immune activation, underscoring the importance of mitochondrial quality control in limiting excessive inflammatory responses and tissue injury.

Collectively, these findings establish mitochondrial metabolism as a central determinant of macrophage function and the pathogenesis of inflammatory disease. Molecular regulators such as mtSTAT3, GPD2, MRPS6, and MAPK14 illustrate how disruptions in mitochondrial bioenergetics, respiratory function, protein synthesis, and quality control converge to reshape macrophage activation and inflammatory signaling. Elucidating these interconnected pathways not only advances our understanding of immune-metabolic regulation but also provides a framework for improving outcomes across a broad spectrum of inflammatory diseases.

## Conclusions

Collectively, the articles published within this Research Topic reinforce the concept of the mito–immune axis as a unifying framework linking immunometabolism, inflammatory pathology and infection. A deeper mechanistic understanding of mitochondrial quality control, metabolic signaling, and organelle–immune crosstalk will not only advance our knowledge of disease pathogenesis but also accelerate the development of mitochondria-directed therapeutics. Targeting mitochondrial pathways, therefore, represents a promising strategy to enhance host defense, restore immune homeostasis, and improve clinical outcomes across a broad spectrum of infectious and chronic inflammatory disorders.

## References

[B7] Chen F. Xu K. Han Y. Ding J. Ren J. Wang Y. . (2024). Mitochondrial dysfunction in pancreatic acinar cells: mechanisms and therapeutic strategies in acute pancreatitis. Front. Immunol. 15, 1503087. doi: 10.3389/fimmu.2024.1503087 39776917 PMC11703726

[B2] Kim M. E. Lim Y. Lee J. S. (2025). Mitochondrial dysfunction and metabolic reprogramming in chronic inflammatory diseases: molecular insights and therapeutic opportunities. Curr. Issues Mol. Biol. 47, 1042. doi: 10.3390/cimb47121042 41614806 PMC12731309

[B6] Klemmensen M. M. Borrowman S. H. Pearce C. Pyles B. Chandra B. (2024). Mitochondrial dysfunction in neurodegenerative disorders. Neurotherapeutics 21, e00292. doi: 10.1016/j.neurot.2023.10.002 38241161 PMC10903104

[B9] Ma R. Zhang C. Liu J. Ren J. Li X. Zhao Y. (2025). Mitochondrial dynamics in autoimmune diseases. Rheumatol. Autoimmun. 5, 258–266. doi: 10.1002/rai2.70011 41531421

[B3] Malla S. Shahreen N. Saha R. (2025). Immunometabolism at the crossroads of infection: mechanistic and systems-level perspectives from host and pathogen. Immunometabolism 7, e00069. doi: 10.1097/in9.0000000000000069 41159060 PMC12558011

[B8] Montgomery M. K. Turner N. (2015). Mitochondrial dysfunction and insulin resistance: an update. Endocrine Connections 4, R1–R15. doi: 10.1530/ec-14-0092 25385852 PMC4261703

[B1] Nakahira K. Hisata S. Choi A. M. (2015). The roles of mitochondrial damage-associated molecular patterns in diseases. Antioxidants Redox Signaling 23, 1329–1350. doi: 10.1089/ars.2015.6407 26067258 PMC4685486

[B4] Phan A. T. Goldrath A. W. Glass C. K. (2017). Metabolic and epigenetic coordination of T cell and macrophage immunity. Immunity 46, 714–729. doi: 10.1016/j.immuni.2017.04.016 28514673 PMC5505665

[B5] Wang Z. He Z. Chang X. Xie L. Song Y. Wu H. . (2025). Mitochondrial damage-associated molecular patterns: new perspectives for mitochondria and inflammatory bowel diseases. Mucosal Immunol. 18, 290–298. doi: 10.1016/j.mucimm.2025.01.013 39920995

